# The Action of Colchicine in Patients with Metabolic Syndrome and Obesity: Perspectives and Challenges

**DOI:** 10.3390/metabo14110629

**Published:** 2024-11-16

**Authors:** Fábio Vieira de Bulhões, Gabriele Eliza Assis, Ana Beatriz Cazé, Jackson Pedro Barros-Pereira, Gabriela Garcia de Carvalho Laguna, Alex Cleber Improta-Caria, Roque Aras-Júnior

**Affiliations:** 1 Faculty of Medicine, Federal University of Bahia (UFBA), Salvador 40110-060, BA, Brazil; fvbulhoes@yahoo.com.br (F.V.d.B.); gabriele.assis@ufba.br (G.E.A.); ana.caze@ufba.br (A.B.C.); jackson.pedro@ufba.br (J.P.B.-P.); 2 Multidisciplinary Institute of Health, Federal University of Bahia (UFBA), Salvador 40110-060, BA, Brazil; gabrielagcl@outlook.com; 3 Laboratory of Biochemistry and Molecular Biology of the Exercise, Physical Education and Sport School, University of Sao Paulo (USP), Sao Paulo 05508-030, SP, Brazil

**Keywords:** cardiometabolic syndromes, abdominal obesity–metabolic syndrome, abdominal obesity, colchicine

## Abstract

Colchicine is an alkaloid traditionally used to treat inflammatory conditions such as gout and familial Mediterranean fever. Currently, there are proposals for the use of this drug in several other situations, such as cardiovascular and liver diseases and diabetes. In this study, the current literature on the potential of colchicine in the treatment of obesity and metabolic syndrome (MS) was evaluated. The inhibitory action of the NLRP3 inflammasome and other processes, such as reductions in the migration and activation of immune system cells, are effects observed in both in vitro studies and animal models related to colchicine, as well as the promotion of mechanisms of the intensification of lipid metabolism, the reduction of tissue fibrosis, and the reduction of serum glucose and triglycerides. These factors are associated with changes in the prognoses of patients with MS, which, together with obesity, has a high association with inflammatory mechanisms for its maintenance and secondary impairments to homeostasis. In humans, clinical research has rarely addressed the use of colchicine in obesity and MS, with only one pilot randomized clinical trial having been conducted, which identified a beneficial anti-inflammatory effect on endothelial function and the process of insulin resistance in this population. However, it is not yet possible to extrapolate its findings and apply its results to a broader context. Given the potential of this “ancient drug” in various pathological contexts and its good tolerability, it is important that its properties continue to be investigated and that more clinical studies be conducted to expand the therapeutic applications of this low-cost substance in patients with obesity and MS.

## 1. Introduction

**Figure 1:**
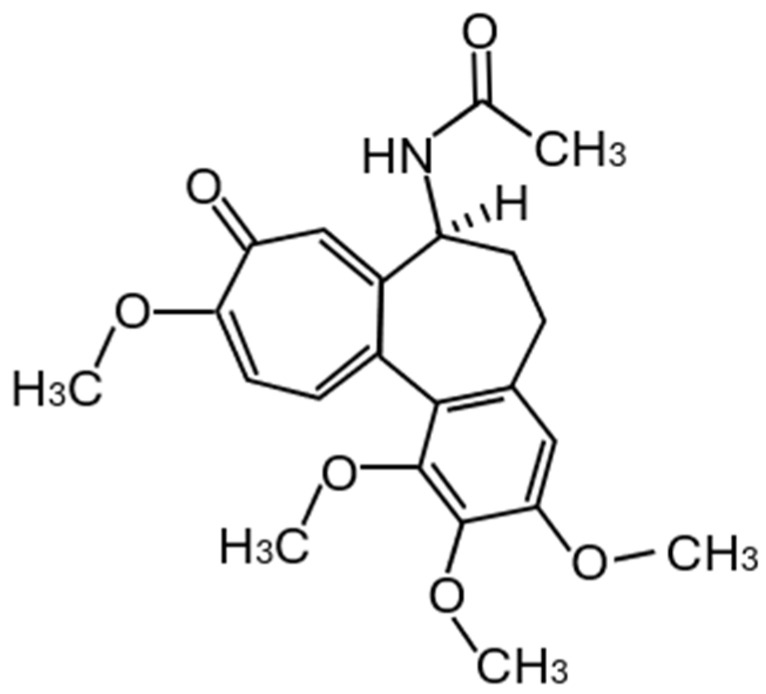
Colchicine molecule C_22_H_25_NO_6_.

Colchicine (Figure 1) is an alkaloid extracted from plants of the genus Colchicum (autumn crocus), mainly from the seeds, flowers, and corms. Autumn crocus is one of the oldest herbal drugs described in human history. It was described in the 1500 BCE Ebers Papyrus of ancient Egypt as being used for joint pain and swelling, and its compound was isolated in the early 1800s and remains in use today as a purified natural product [1,2,3]. Today, the therapeutic use of colchicine is related to inflammatory and fibrotic conditions.

Colchicine is used to treat gout, familial Mediterranean fever (FMF), Behcet’s disease, pericarditis, osteoarthritis, and coronary artery disease [4]. This drug acts in different anti-inflammatory pathways by inhibiting proinflammatory cytokines, as well as inflammasome and neutrophil migration [5]. Moreover, colchicine can prevent the release of inflammatory glycoproteins and phagocytes, which may interfere with the inflammatory process related to adipose tissue [6,7]. New drugs are gaining ground in the treatment of obesity and metabolic syndrome and Colchicine may be an important tool.

Metabolic syndrome (MS) is a complex multifactorial disorder characterized by abdominal obesity, insulin resistance, high blood pressure (HBP), and atherogenic dyslipidemia [8,9,10]. MS can affect up to 2.4 billion people worldwide and is associated with the global cardiovascular crisis and reduced life expectancy [8,9,10,11]. Over the past 20 years, the prevalence of all metabolic diseases has increased, with the greatest growth occurring in countries with high socioeconomic indexes [12].

The risk factors for MS include genetic predispositions, sedentary lifestyles, increased caloric intake, and environmental factors [13]. Obesity is considered the main component of MS, as it can lead to insulin resistance, HBP, and dyslipidemia [4,14]. The excess of macronutrients in adipose tissue triggers the release of inflammatory mediators such as tumor necrosis factor-alpha (TNF-α) and interleukin 6 (IL-6). Moreover, adipose tissue reduces adiponectin production, predisposing individuals to a proinflammatory state and oxidative stress [15].

Although the mechanism of the action of colchicine is well described in the literature, its effect on metabolic syndrome and obesity is not yet well established. This narrative review aims to describe the potential benefits of colchicine in the treatment of obesity and MS.

## 2. Mechanisms of Action of Colchicine

**Figure 2:**
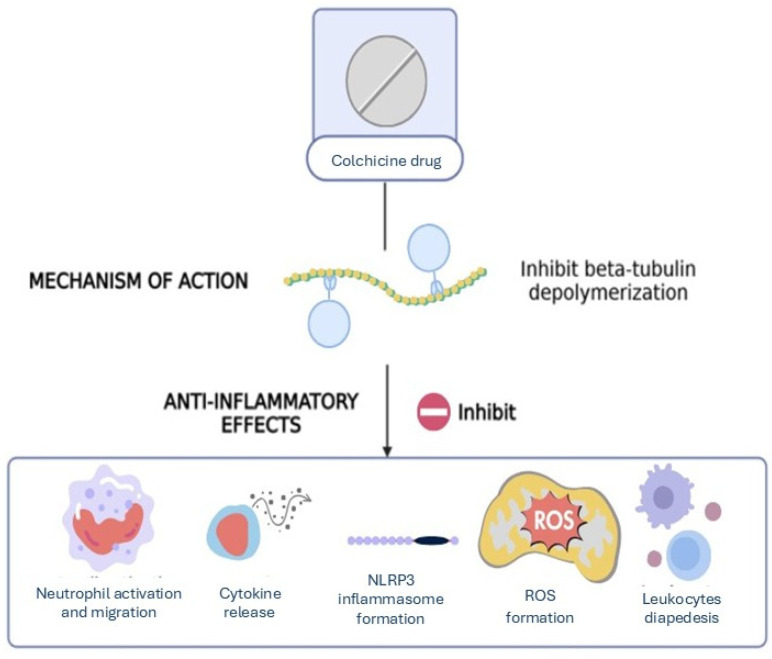
Mechanism of action of colchicine and its anti-inflammatory effects.

Colchicine exerts its anti-inflammatory effects by preventing tubulin polymerization, which disrupts microtubule formation and impairs the cytoskeletal functions necessary for cell division, migration, and intracellular transport [16,17,18]. This disruption affects several cellular processes, including the function of neutrophils and the activation of the NLRP3 inflammasome [16,17,18,19]. The prevention of the polymerization of β-tubulin inhibits the activation, degranulation, and migration of neutrophils to sites of inflammation [19]. In addition, this process triggers a cascade of events, which inhibits the following: (1) locomotion and diapedesis of leukocytes; (2) cytokine production; (3)formation of reactive oxygen species (ROS); and (4) formation of the NLRP3 inflammasome, a key component in the chronic inflammation observed in obesity [20,21] (Figure 2).

Colchicine can bind to tubulin proteins, effectively blocking the polymerization of microtubules. Microtubules are structures formed by α- and β-tubulin heterodimers and are key components of the cellular cytoskeleton. They are responsible for maintaining cell shape, facilitating intracellular transport, and regulating the secretion of cytokines and chemokines. Colchicine’s interaction with tubulin can reduce inflammatory responses as it inhibits inflammatory cell recruitment and activation [2,3,4,5,6,7,8,9,10,11,12,13,14,15,16]. In addition, colchicine functions as an antimitotic agent. This antimitotic effect has implications beyond inflammation management, as research increasingly explores colchicine’s potential as an anti-tumor therapy [22,23,24].

Colchicine causes the inhibition of neutrophil extracellular traps (NETs), neutrophil chemotaxis, and the release of a glycopeptide crystal-derived chemotactic factor from neutrophil lysosomes after phagocytosis of monosodium urate crystals [24,25,26,27]. Moreover, colchicine can reduce oxidative stress by reducing calcium influx into neutrophils. Colchicine also suppresses monosodium urate (MSU)-induced NALP3 inflammasomes, which are responsible for caspase-1 activation and the subsequent IL1β and IL18 processing and release. This could possibly be related to the disruption of the microtubule-dependent transport of mitochondria to the endoplasmic reticulum [16].

## 3. The Role of Inflammatory Mechanisms in Obesity and Metabolic Syndrome

The underlying inflammatory mechanisms interconnecting obesity with metabolic dysfunction are not completely understood. Obesity and metabolic syndrome are strongly associated with a state of low-grade chronic inflammation that affects the entire body [28,29,30]. Adipose tissue (AT) can be classified into brown adipose tissue (BAT) and white adipose tissue (WAT). WAT represents a major factor in the development of cardiovascular conditions and complications, while BAT constitutes only a minor proportion of AT, playing anti-obesity and anti-diabetic roles, and is involved in thermogenesis [31,32,33]. Adipocytes not only function as lipid storage, but also act as dynamic endocrine organs, secreting various bioactive molecules such as leptin and adiponectin [15].

Leptin is a satiety hormone and has pro-inflammatory effects, whereas adiponectin exhibits anti-inflammatory properties by downregulating inflammatory molecules. Importantly, serum levels of adiponectin decrease significantly with weight gain and obesity [29]. The adipocyte hypertrophy that occurs in obesity leads to cellular dysfunction and adipocyte death. This process results in the release of danger signals that recruit macrophages and other immune cells to the adipose tissue, exacerbating local and systemic inflammation [15,34]. Macrophages, predominantly of the M1 type, secrete the following: (1) proinflammatory cytokines, such as TNF-α, IL-6, and IL-1β; (2) proinflammatory adipokines, such as leptin, plasminogen activator inhibitor type 1 (PAI-1), visfatin, and resistin; and (3) inducible nitric oxide synthase (iNOS) [35].

An inflammasome is a large protein complex that acts as a sensor in innate immune cells, detects diverse harmful signals, and activates caspase-1, which triggers the secretion of potent pro-inflammatory cytokines [36]. In obesity the activation of the NLRP3 inflammasome promotes the recruitment and activation of macrophages in adipose tissue, which in turn stimulates T cell activation. This cascade of immune responses leads to chronic inflammation, perpetuating a pro-inflammatory environment that disrupts insulin signaling, further impairing insulin sensitivity in key metabolic tissues such as the liver and skeletal muscle [30,36].

Therefore, therapeutic strategies targeting inflammation, particularly the pathways involved in caspase-1 activation—a key component of inflammasome activity—could represent a potential strategy to mitigate the chronic inflammation associated with obesity and improve insulin sensitivity [30].

## 4. Investigating Colchicine in Obesity and Metabolic Syndrome: Insights from In Vitro and Animal Models

### 4.1. Colchicine Stimulates Browning of White Adipocytes

Choi et al. [37] studied a white adipocyte culture and found that colchicine stimulated white adipocyte browning in 3T3-L1 white adipocytes mainly through antagonism of GABA-BR and agonism of β3-AR, a fat-specific marker of these adipocytes. Therefore, the mechanistic study showed that colchicine induced browning by elevating the thermogenic marker genes and proteins and improved the lipid catabolic metabolism [37].

### 4.2. Metabolic Parameters and Inflammatory Responses in Rodent Models

A study conducted on rodent models with obesity (Sprague-Dawley males) revealed that colchicine can improve metabolic parameters and inhibit inflammatory responses. In addition, it showed a cardiac impact by reducing ventricular fibrosis; increasing the expression of Cav1.2, Kv4.2, Nav1.5, and Cx43; and reducing the expression of CaMKII, p-CaMKII, p-RyR2 (S2808), and p-RyR2 (S2814) in the left ventricle. In addition, colchicine prolongs the ventricular effective refractory period (ERP), reducing the corrected QT interval (QTc) and the Tpeak-Tend interval. Thus, the medication was able to reduce susceptibility to ventricular arrhythmias and was able to lead to a reduction in body weight [38].

### 4.3. Colchicine Attenuating Cholesterol Levels in Rabbits Models

A study conducted on twenty-three male New Zealand White rabbits, divided into three groups and fed different types of diet for 7 weeks—standard, cholesterol 1% *w*/*w*, and cholesterol 1% *w*/*w* plus colchicine 2 mg/kg of body weight—revealed that the colchicine group had decreased triglyceride (TG) levels at the end of the study (*p* < 0.05), while the group that received only cholesterol had increased levels. In addition, colchicine acts as a hypoglycemic drug, reducing plasma glucose levels. However, in this study, colchicine did not affect the development of atherosclerotic plaques, appearing to favor the creation of fatty streaks in the aorta of rabbits, since the thickness of the intima in the ascending and thoracic aorta of the cholesterol and colchicine group was significantly greater than that of the other groups (*p* < 0.05). In addition, no statistical differences were observed in the levels of IL-18, leptin, and insulin between the groups. Thus, in summary, colchicine administration did not influence the progression of atherosclerosis or the IL-18, insulin, and leptin levels in rabbits fed cholesterol; however, it played an attenuating role in their TG and plasma glucose levels [39].

## 5. Clinical Research on Colchicine in Humans

### 5.1. Effects of Colchicine on Metabolic Outcomes in People with Obesity and/or Metabolic Syndrome

Only one single-center clinical trial has been conducted to analyze colchicine and its effects on obesity and metabolic syndrome. A total of 40 patients were randomly assigned to either the intervention group (IG), with 21 participants receiving 0.6 mg of colchicine two times a day for 3 months, or to the control group (CG), with 19 participants receiving a placebo two times a day for 3 months. In total, 92.5% (37/40) of the patients completed the study. The results of the primary and secondary analyses revealed that colchicine was able to reduce inflammation and insulin resistance, improve endothelial cell function, and improve atherosclerosis in obese individuals [40]. The results of this study used the same population and were published in different articles, as shown in Table 1.

Some results that we can highlight are that colchicine:

1. Reduces systemic markers of inflammation, such as high-sensitivity CRP; erythrocyte sedimentation rate (ESR); white blood cell and leukocyte count; absolute neutrophil count; and levels of hsCRP, GlycA, IL-6, and resistin [21,39,41].
2. Affects the circulating leukocyte populations involved in the innate and adaptive immune systems and the associated inflammatory secretome through reductions in total monocytes, innate leukocytes, NK cells, CD4+ effector T cells, CD8+ cytotoxic T cells and increased dendritic cells, lymphoid progenitor cells, central memory TCD4+, central memory TCD8+ cells, and high CD8+CD38 cells [40].
3. Does not alter insulin sensitivity; however, there are changes in HOMA-IR, fasting insulin, and glucose efficacy that suggest metabolic improvements in the colchicine group [39].
4. Reduces biomarkers of systemic inflammation that appear to improve insulin’s regulation of lipolysis, with a decrease in the rates of insulin-suppressible lipolysis and maximal lipolysis [42].
5. Does not alter leukocyte population distributions in subcutaneous adipose tissue, nor is there evidence that colchicine improves circulating atherogenic lipoprotein particle concentrations [21,42].
6. Has no effects on measurements of gut microbiome alpha diversity [41].

### 5.2. Changes in Body Weight and BMI Caused by Colchicine

Inflammation is one of the main factors related to metabolic dysregulation in obese patients. Colchicine inhibits the activation of the NLRP3 inflammasome, a multiprotein complex associated with the secretion of inflammatory cytokines such as interleukin-1 beta (IL-1β). This protein complex can contribute to the development of insulin resistance and obesity. So inhibition of the NLRP3 inflammasome could have a beneficial effect on patients by improving insulin sensitivity and promoting weight loss [20].

Research on the effects of colchicine on weight loss is still limited. There is only one pilot study that has shown that colchicine can reduce the presence of inflammatory drugs and improve metabolic and cardiovascular outcomes in adults with obesity and MS. However, it did not identify significant effects on weight loss or BMC reduction [40,41,42].

### 5.3. Colchicine in Chronic Liver Disease: A Proxy for MS

Despite the lack of studies in patients exclusively with MS and/or obesity, a safety analysis with MS-related diseases has shown significant results for the use of colchicine. Previous studies have demonstrated the role of colchicine in reducing fibrosis in patients with mild to moderate liver cirrhosis, regardless of the cause [45]. In a randomized clinical trial by Nikolaidis et al. [46] treatment was performed for at least 12 months in patients with chronic liver disease (chronic hepatitis C and fatty liver disease). There was an increase in the CD4:CD8 ratio of peripheral blood T lymphocytes and in serum albumin levels. In contrast, after 24 months, patients treated with colchicine showed a reduction in their serum levels of aminoterminal procollagen peptide III (PIIINP), a valuable index that estimates fibrogenesis [46]. Therefore, the use of colchicine is well tolerated by patients and the drug can exert anti-inflammatory, antifibrotic, and immunomodulatory effects. In view of these effects, the author hypothesized that long-term use of colchicine could also be beneficial for individuals with MS [47].

### 5.4. Colchicine and the HOMA-IR Index, Blood Glucose, and Glycated Hemoglobin

Systemic inflammation is closely related to the pathogenesis of type 2 diabetes mellitus (T2DM). Insulin resistance results in increased visceral adiposity and nutrient overload, with inflammation being an important etiological factor in this complex process [48]. People with T2DM often have elevated levels of inflammatory markers, such as CRP, IL-6, and TNF-a [49].

Hyperglycemia in patients with T2DM induces an increase in reactive oxygen species (ROS) and ROS-dependent activation of the NLRP3 inflammasome. NLRP3 activation is associated with metabolic distress, inflammation, and the development of insulin resistance. Thus, the inhibition of NLRP3 inflammasome activation shows promise in preventing the progression of insulin resistance to T2DM [48,49,50].

Some studies have evaluated the relationship between colchicine use and the risk of T2DM. A cohort study conducted by Chu et al. [51] with 3841 gout patients who used colchicine and 7682 gout patients who did not use colchicine demonstrated that the risk of T2DM was significantly lower in colchicine users than in nonusers. Moreover, this study found that the inverse relationship between colchicine use and the risk of diabetes remained consistent across sexes and age groups [51].

A study conducted in a population with DM2 reported favorable results with colchicine. This clinical trial included 150 newly diagnosed DM2 patients, randomized into three parallel groups: (1) colchicine (0.5 mg twice daily); (2) metformin (1 mg per day); and (3) diet instructions. This study demonstrated that both metformin and colchicine reduce A1C with a *p*-value < 0.001 [52]. Demidowich [40] found a relationship between the use of colchicine and improvement in the HOMA-IR index, and a trend toward improvement in fasting insulin and glucose efficacy, suggesting metabolic improvements in this group [39].

### 5.5. Colchicine for the Prevention of Cardiovascular Events

Colchicine reduces cardiovascular events by inhibiting the IL-1β/IL-6/CRP inflammatory pathway, which is involved in the progression and rupture of atherosclerotic plaques. By decreasing the release of these cytokines, the drug stabilizes plaques, reducing the risk of cardiovascular complications [53]. Colchicine is also capable of reducing vascular inflammation and can be used as a therapy for coronary artery disease (CAD) in the secondary prevention of acute myocardial infarction (AMI) and stroke [54].

Despite the fact that dyslipidemia, DM, and MS are risk factors for cardiovascular diseases, they are not the only ones responsible for the atherosclerosis process. Recently, it has been recognized that the inflammatory pathway in atherosclerotic cardiovascular disease (ASCVD) is also influenced by the immune system [55]. This process occurs through the innate and adaptive immune systems. In addition, several cytokines, such as IL-1, IL-12, IL-18, TNF-α, MIF, IFN-γ, and M-CSF, have shown proatherogenic effects [56].

The studies COLCOT [57] and LODOCO2 [58] found that colchicine is an anti-inflammatory therapy capable of reducing the risk of adverse cardiovascular events in patients with previous CAD. This drug inhibits the production of cytokines by blocking tubulin polymerization and inhibiting adhesion molecules such as NLRP3. NLRP3 is essential for caspase-dependent cleavage 1 and the subsequent release of the proinflammatory cytokines IL-1β and IL-18. These agents are more effective in those with high systemic inflammation [59]. In June 2023, the US Food and Drug Administration (FDA) approved the use of 0.5 mg of colchicine once daily to reduce cardiovascular events in patients with established ASCVD and high residual systemic inflammation, defined as a CRP level ≥ 2 mg/L [60].

## 6. Adverse Events Associated with Colchicine

### 6.1. Adverse Events in Obese Populations and Metabolic Syndrome

Few studies have analyzed the use of colchicine in obese individuals or those with MS. Currently, we only have the results of a pilot study in which the rates of adverse events were not significantly different between the groups. In this study, most adverse events were classified as mild (Grade 2 or less). Adverse events of the gastrointestinal tract prevailed (IG = 13; CG = 14), followed by those of the upper respiratory tract (IG = 6; CG = 11), fatigue (IG = 6; CG = 5), headache (IG = 3; CG = 4), neurological signs (IG = 3; CG = 3), and skin irritation (IG = 2; CG = 2) [39].

The adverse effects in the control group were as follows: one patient developed a case of anemia; one developed both leukopenia and neutropenia; five experienced creatine kinase increases; three developed elevated AST; two developed elevated ALT; and one subject developed a Grade 3 transaminitis (ALT elevation), which improved spontaneously. In the intervention group, the adverse effects were as follows: one participant developed both leukopenia and neutropenia; two developed thrombocytopenia; three experienced creatine kinase increases; four developed elevated ALT; and five subjects developed elevated AST. All adverse effects improved/resolved spontaneously during or after the study intervention. Thus, the study demonstrated that colchicine is well tolerated by adults with obesity and MS [39].

### 6.2. Long-Term Use in Familial Mediterranean Fever and Cardiovascular Disease

Clinical trials involving the long-term use of colchicine in individuals with familial Mediterranean fever and cardiovascular diseases did not identify serious adverse events. The most prevalent ones were gastrointestinal effects, including diarrhea, nausea, and vomiting [57,58,61,62]. A small percentage of patients (10.8%) undergoing long-term therapy had reversible leukopenia [63]. Bleeding complications are rare but may occur due to colchicine’s antiplatelet effects [61,64]. In some studies, a potential increase in pneumonia and infections has been demonstrated in groups that used colchicine, which may reflect altered immune responses [57,65].

## 7. Contraindications and Drug Interactions

### 7.1. Renal Function

Colchicine has a relative contraindication in people with CrCL < 60 mL/min and the dose should be adjusted in patients with mild renal impairment [66].

### 7.2. Drug Interactions

Colchicine has potential drug interactions with other medications, such as statins and calcium channel blockers. Colchicine is metabolized in the liver by demethylation and its metabolism can compete for the CYP3A4 isoenzyme. This process can lead to higher serum concentrations of drugs, thereby increasing the risk of side effects. Colchicine and statin-induced myopathy have been reported, and some case reports correlate the concomitant use of the two classes with the onset of myopathies and rhabdomyolysis [67,68,69,70,71,72].

## 8. Previous and Ongoing Clinical Trials on the Use of Colchicine in Metabolic Syndrome, Obesity, and Diabetes

The protocols for clinical trials related to colchicine use for metabolic syndrome and/or obesity are shown in Table 2.

## 9. Discussion

Colchicine is an anti-inflammatory agent that acts in inflammatory pathways by interfering in the cell microtubules’ assembly, reducing the neutrophil activity, and suppressing cytokine production and inflammasome NLRP3 formation [1,2,3,4,5,6,7,8,9,10,11,12,13,14,15,16,17,18,19,20,21,53]. It is widely used in diseases related to inflammation, such as gout, and is currently being used in the reduction of vascular inflammation, the stabilization of atherosclerotic plaques, and the secondary prevention of cardiovascular events, such as AMI and stroke [54,74]. Obesity and MS are closely related to inflammation. The use of colchicine as a possible treatment to control inflammatory activity has not yet been thoroughly studied in humans, with only one pilot clinical trial completed to date [15,28,29,30,35,36,37,38,39,40]. Here, we highlight the potential of this drug based on the colchicine mechanism of action and provide an overview of the current literature on the subject.

Despite the limited number of studies on the use of colchicine in MS and obesity, this drug has been extensively studied in other conditions. The findings show secondary benefits for diseases related to obesity and MS, such as diabetes mellitus, cardiovascular diseases, and chronic liver disease [75]. Benefits include reduced hepatic fibrosis in cirrhotic patients, reduced hemoglobin glycation in DM2, and lower rates of cardiovascular events linked to atherosclerotic plaques [46,47,52,53]. We hypothesized that colchicine could have a great impact on obesity and MS due to its anti-inflammatory effects, especially in the NLRP3 inflammasome, a key component of chronic inflammation in obese patients [20,21].

In recent years, several studies have investigated the properties of colchicine, suggesting a promising effect on glycemic control. These studies indicate that colchicine may reduce levels of blood glucose, glycated hemoglobin, and inflammatory markers such as CRP, demonstrating its potential for the treatment of T2DM, which is characterized by chronic inflammation [76,77,78]. Additionally, the research suggests that colchicine use may contribute to lowering the risk of developing T2DM. There is a reinforced need for further studies on this topic, to better understand these mechanisms and effects, as well as to establish safe protocols for its use in this context [51,79,80].

In the context of diabetes management, metformin remains the initial choice for glycemic control in type 2 diabetes, due to its efficacy and safety. In addition to controlling blood glucose, newer pharmacological agents like GLP-1 receptor agonists and SGLT2 inhibitors offer added benefits, such as significant weight loss and reduced cardiovascular risk [77].

The treatment of obesity follows the guidelines of the American College of Cardiology (ACC), the American Heart Association (AHA), and The Obesity Society (TOS), published in 2014, and of the American Association of Clinical Endocrinologists (AACE) and the American College of Endocrinology (ACE), published in 2016 [81]. Sometimes, lifestyle changes are not enough to help patients lose height. Therefore, drugs approved by the FDA can be used [81]:

1. GLP-1 receptor agonist—semaglutide and liraglutide;
2. Combination sympathomimetic amine anoretic/anti-epileptic analogue—phentermine HCl and topirame extended-release capsules;
3. Combination opiod antagonist/aminoketone antidepressant—naltrexone HCL and bupropion HCl extended-release tablets;
4. Lipase inhibitor—Orlistat.

In addition to the potential biological benefits of weight loss, it should be remembered that obesity is a multifactorial condition that involves many aspects such as mental health, socioeconomic status, culture, and access to health services. Therefore, studying accessible and low-cost medications, such as colchicine, is essential to provide patients with tools to treat this condition [82,83].

Studies carried out on animals with obesity and MS treated with colchicine have shown that it provides benefits such as reduced inflammation and ventricular fibrosis, modulation of cardiac protein expression, decreased susceptibility to arrhythmias, and weight loss [38]. There have been different findings in animal models compared to humans, as the work of Kaminiotis et al. [39] did not find any reduction in cytokines or atherosclerotic plaques [39]. This shows the importance of new randomized controlled trials with humans to analyze the real benefits and side effects of colchicine.

In a study analyzed here, colchicine administered twice daily for 3 months improved insulin resistance and endothelial function, and also modulated leukocytes and inflammatory secretome [21,41,42,44]. This could be a potential drug target for the treatment of obesity and MS with low side effects and a low cost for public health systems worldwide. Due to the epidemic of obesity and MS it is crucial to understand and to improve the treatment of these diseases that have a high morbimortality.

Colchicine is generally well tolerated, with mild adverse events that are mostly gastrointestinal, and no serious adverse events reported. The efficacy and safety of colchicine in patients with significant renal impairment (CrCL < 60 mL/min) require further attention. In addition, special attention should be given to its drug interaction with statins and calcium channel blockers [57,78]. This reinforces the need for more robust studies to elucidate long-term safety and to validate colchicine as an effective therapeutic option for obesity and MS.

## 10. Conclusions

This study summarizes the inflammatory modulating effect of colchicine, with its reduction of inflammatory biomarkers and improvement of endothelial function in patients with MS. However, the data present in the literature are still mostly limited to studies in animal models and in vitro, with only one pilot study focusing on investigating the true action of this drug in a population with MS. Considering the high rate of obese patients and MS in the global population, as well as the impact of these conditions on individuals, the search for an accessible and effective therapy should be a priority. Therefore, it is essential to continue investigating the effects of colchicine in a clinical context to guide practical decision-making and clarify the benefits, risks, and long-term effects of its use in the management of these conditions. Therefore, the need for subsequent studies in human populations to verify these potential benefits is demonstrated. Future randomized controlled trials with larger sample sizes and population diversity have the potential to strengthen the current evidence, being able to investigate in greater detail the outcomes of BMI reduction and the improvement of glycemic indicators and HBP, as well as their safety in patients with renal comorbidities.

## Figures and Tables

**Table 1:** The results of the pilot study carried out in a population with metabolic syndrome and/or obesity *.

Author, Year	Summary of the Effects Associated with the Use of Colchicine Compared with Placebo
Patel et al., 2023 [41]	Colchicine reduced the levels of NK cells (*p* = 0.002) and reduced the inflammation biomarkers COX2 (*p* = 0.002 for colchicine group), SPD (*p* = 0.017 for colchicine group), myeloperoxidase (*p* = 0.050 for colchicine group), proteinase-3 (*p* = 0.042 for colchicine group), IL-16 (*p* = 0.064 for colchicine group), resistin (0.026 for colchicine group), and phosphodiesterase 5A (*p* = 0.007 for colchicine group). In dendritic cells, colchicine was associated with alterations in serum concentrations of hFABP; reductions in CD4+ effector T cells (*p* = 0.031) and CD8+ cytotoxic T cells (*p* = 0.019); increases in CD4+ (*p* = 0.036) and CD8+ (*p* = 0.004) central memory T cells; and activation of CD38 High CD8+ T cells (*p* = 0.004).
Kisimba et al., 2023 [42]	Despite significantly reducing the levels of systemic inflammation (*p* < 0.02), colchicine had no significant effect on the measurements of alpha diversity in the gut microbiome. *Oscillibacter* was the only genus to show a nominally significant reduction (*p* = 0.04).
Levine et al., 2022 [43]	Decreased rates of insulin-suppressible lipolysis and maximal lipolysis correlated with a reduction in biomarkers of systemic inflammation (CRP, resistin, circulating monocytes, and neutrophils) (*p* < 0.05). Colchicine did not significantly alter leukocyte population distributions in the SAT (*p* > 0.05).
Demidowich et al., 2020 [44]	Colchicine decreased concentrations of the inflammatory molecules CRP, IL-6, and resistin, as well as vascular proteins such as the oxidized low-density lipoprotein receptor and phosphodiesterase 5 A (*p* < 0.01). It increased the concentrations of factors associated with metabolism, as well as hFABP (hepatocyte growth factor activator) and the antithrombotic molecule protein C.
Demidowich, et al., 2019 [21]	Colchicine reduced the inflammation markers CRP, erythrocyte sedimentation rate, and GlycA (*p* < 0.005). LDL-ox and small LDL concentrations increased in the colchicine arm (*p* = 0.022). There was no significant effect on other lipoprotein subfractions or lipoprotein particle sizes (*p* > 0.05).
Demidowich AP et al., 2019 [40]	Colchicine reduced high-sensitivity CRP, ESR, white blood cell count, leukocyte count, and absolute neutrophil count (*p* < 0.005). The change in insulin sensitivity was not significantly different between the groups (*p* = 0.082). However, changes in the secondary results of HOMA-IR, fasting insulin, and glucose efficacy suggested metabolic improvements in the colchicine group.

Footer: NK = Natural Killer; COX-2 = cyclooxygenase 2; SPD = surfactant protein D; IL-16 = interleukin 16; IL-6 = interleukin 6; hFABP = heart-type fatty acid-binding protein; CRP = C-Reactive Protein; SAT = subcutaneous adipose tissue; GlycA = glycoprotein acetylation, which refers to an inflammatory marker; oxLDL = oxidized low-density lipoprotein; LDL = low-density lipoprotein; ESR = erythrocyte sedimentation rate; HOMA-IR = Homeostatic Model Assessment of Insulin Resistance. * All data from the above studies come from the same pilot clinical trial that evaluated 40 adults with obesity and metabolic syndrome, consisting of an intervention group that received colchicine and a placebo group that received no intervention. The study was conducted in the city of Bethesda, Maryland, between 2014 and 2018.

**Table 2:** Current clinical trials on the use of colchicine in patients with metabolic syndrome published by ClinicalTrials.gov [73] through August 2024.

Title	Study Identification	Status	Objectives
The Effect of Colchicine on Food-related Effort-based Decision Making in Brain and Behaviour in Overweight and Obesity (FLAIR-i)	NCT05785429	Recruitment	Analyze the role of inflammation in effort-based decision-making in the brain and in the behavior of overweight and obese people, comparing the effect of colchicine with a placebo.
Repurposing Colchicine for Reduction of Residual Inflammatory Risk in Type 1 Diabetes (REC1TE)	NCT05949281	Recruitment	Assess whether colchicine, in addition to standard treatment, improves the markers of inflammation and cardiovascular disease in people with T1DM. Participants will be assigned a daily 0.5 mg dose of colchicine or placebo at a 1:1 ratio for 26 weeks.
Colchicine and Non-enteric Coated Aspirin in the Cardiovascular Outcomes Trial of Patients With Type 2 Diabetes (COLCOT-T2D)	NCT05633810	Recruitment	Evaluate the efficacy and safety of colchicine and non-enteric coated aspirin, combined or alone, to improve cardiovascular outcomes in high-risk patients with T2DM.
Colchicine to Suppress Inflammation and Improve Insulin Resistance in Adults and Adolescents With Obesity	NCT05017571	Recruitment	Analyze whether colchicine can improve metabolism in people with high body weight, increased inflammation, and high insulin in the blood, but who have not yet developed DM.
Effects of Colchicine in Non-Diabetic Adults With Metabolic Syndrome	NCT02153983	Completed	Evaluated whether colchicine improves sugar regulation and metabolism.
Colchicine has Anti-diabetic Effect	NCT04377321	Completed	Assessed colchicine’s effect on the A1C of newly diagnosed T2DM compared to metformin and a control group treated through diet only.
Dose-finding Study of Colchicine in Type 2 Diabetic Patients With Coronary Artery Disease (DRC-04)	NCT03376698	Completed	Investigated the dose-dependent effects of colchicine on inflammatory responses and endothelial function in T2DM with CAD and leukocyte activation.
Low-dose Colchicine in Patients With Type 2 Diabetes Mellitus and Microalbuminuria	NCT02035891	Unknown status	Avaliated whether low-dose colchicine slows the progression of microvascular complications in patients with T2DM and microalbuminuria who have recived stable treatment of ACEI/ARB for at least 3 months

DM, diabetes mellitus; T1DM, type 1 diabetes mellitus; T2DM, type 2 diabetes mellitus; CAD, coronary artery disease; ACEI/ARB, angiotensin-converting enzyme inhibitor/angiotensin II receptor blocker.

## Data Availability

No new data were created or analyzed in this study. Data sharing is not applicable to this article.
